# Structural Basis for Inhibition of the MDM2:p53 Interaction by an Optimized MDM2-Binding Peptide Selected with mRNA Display

**DOI:** 10.1371/journal.pone.0109163

**Published:** 2014-10-02

**Authors:** Takashi Nagata, Kie Shirakawa, Naohiro Kobayashi, Hirokazu Shiheido, Noriko Tabata, Yuko Sakuma-Yonemura, Kenichi Horisawa, Masato Katahira, Nobuhide Doi, Hiroshi Yanagawa

**Affiliations:** 1 Institute of Advanced Energy, Kyoto University, Gokasho, Uji, Kyoto, Japan; 2 Graduate School of Energy Science, Kyoto University, Gokasho, Uji, Kyoto, Japan; 3 Department of Biosciences and Informatics, Keio University, Yokohama, Kanagawa, Japan; 4 Institute for Protein Research, Osaka University, Suita, Osaka, Japan; University of Essex, United Kingdom

## Abstract

The oncoprotein MDM2 binds to tumor suppressor protein p53 and inhibits its anticancer activity, which leads to promotion of tumor cell growth and tumor survival. Abrogation of the p53:MDM2 interaction reportedly results in reactivation of the p53 pathway and inhibition of tumor cell proliferation. We recently performed rigorous selection of MDM2-binding peptides by means of mRNA display and identified an optimal 12-mer peptide (PRFWEYWLRLME), named MDM2 Inhibitory Peptide (MIP), which shows higher affinity for MDM2 (and also its homolog, MDMX) and higher tumor cell proliferation suppression activity than known peptides. Here we determined the NMR solution structure of a MIP-MDM2 fusion protein to elucidate the structural basis of the tight binding of MIP to MDM2. A region spanning from Phe^3^ to Met^11^ of MIP forms a single α-helix, which is longer than those of the other MDM2-binding peptides. MIP shares a conserved Phe^3^-Trp^7^-Leu^10^ triad, whose side chains are oriented towards and fit into the hydrophobic pockets of MDM2. Additionally, hydrophobic surface patches that surround the hydrophobic pockets of MDM2 are covered by solvent-exposed MIP residues, Trp^4^, Tyr^6^, and Met^11^. Their hydrophobic interactions extend the interface of the two molecules and contribute to the strong binding. The potential MDM2 inhibition activity observed for MIP turned out to originate from its enlarged binding interface. The structural information obtained in the present study provides a road map for the rational design of strong inhibitors of MDM2:p53 binding.

## Introduction

Tumor suppressor protein p53 plays a crucial role in maintaining genetic stability and preventing cancer formation [1]. p53, a transcription factor whose expression level increases in response to cellular stress such as DNA damage, transactivates various target genes that are involved in antitumor activities, as exemplified by p21^WAF1/CIP1^ (cell-cycle arrest), and Bax and Puma (induction of apoptosis) [2]–[4]. Thus, inactivation of p53 leads to accumulation of genetic aberrations that may cause upregulation of several kinds of oncoproteins, resulting in tumorigenesis [5]. In approximately half of all human cancer, p53 is inactivated by mutations, whereas in the rest, p53 is functionally inhibited by negative regulators, of which the best known is MDM2 [6]–[8].

MDM2 is an E3 ubiquitin ligase that inactivates p53 by directly binding to an intrinsically disordered region of its N-terminal transactivation domain. MDM2 promotes nuclear export of p53, by which the expression of p53-regulated genes is suppressed [9], [10]. In other cases, MDM2 recruits E2 ubiquitin-conjugating enzymes to ubiquitinate p53, resulting in proteasomal degradation of p53 [7], [11]–[13]. MDMX, a homolog of MDM2 that lacks E3 ubiquitin ligase activity, binds to the same region of p53 as MDM2 and thereby negatively regulates p53 [14]. It has been shown that abrogation of the MDM2:p53 interaction leads to reactivation of the p53 pathway and inhibition of tumor cell proliferation [15], [16].

Several small-molecular compounds and peptides mimicking the MDM2 binding site of p53 have been reported to inhibit the MDM2:p53 interaction, antagonizing MDM2 and activating the p53 pathway in cancer cells [14], [17]–[19]. The crystal structure of the MDM2:p53 complex revealed that the region spanning amino acid residues 15–29 of p53 (p53^15–29^) is important in binding to MDM2, and residues F19 to L26 form an amphiphilic α-helix in the complex, in which the side chains of F19, W23, and L26 (Phe-Trp-Leu triad) dock inside the hydrophobic pockets of MDM2 [20]. The crystal structures of peptide antagonists against MDM2 in complexes with MDM2 showed that this docking of the Phe-Trp-Leu triad is conserved [21]. The crystal structures of small-molecule antagonists in complexes with MDM2 showed that the Phe-Trp-Leu triad is replaced by simple hydrophobic functionalities, which fill the hydrophobic pockets of MDM2 [22]. Therefore, one possible approach for the discovery of better MDM2 binders would be the exploration of additional possible interactions.

Generally, peptides are more robust tools for disrupting protein-protein interactions compared to small-molecules since their large interacting surfaces confer higher specificity and affinity, resulting in fewer adverse side effects when applied as pharmaceutical agents. We recently performed *in vitro* selection of MDM2-binding peptides [23] from random peptide libraries using the *in vitro* virus (mRNA display) method [24], [25]. This system, based on cell-free translation, is a potent method for the screening of functional peptides [26], [27] and proteins [28]–[30] from large-sized libraries (∼10^13^ unique members), which exceed the sizes of libraries covered by phage display. We divided the mRNA display screening procedure into two stages, the size of the search space being reduced in the second stage according to the solution of the first stage, to perform a complete search efficiently. As a result, we identified an optimal 12-mer peptide (PRFWEYWLRLME), which was named MIP [23].

We recently showed that (i) MIP inhibits the MDM2:p53 interaction in living cells and thereby blocks tumor cell growth, and (ii) MIP exhibits a higher affinity for MDM2 (and MDMX) and higher tumor cell proliferation suppression activity than known peptides, such as DI [14]. Here, we report investigation of the MIP:MDM2 interaction through NMR structure determination to better understand the origin of the MIP's optimized binding and functional characteristics.

## Materials and Methods

### Construction of expression vectors

First, a DNA fragment encoding a HAT-GB1-MIP-TEV cleavage site was generated as follows. Two oligonucleotides, 5′- TATGCCCAGGTTCTGGGAGTACTGGTTGCGGTTAATGGAGGACTACAAGGACGATGACGACAAGTAATAG-3′ and 5′-GATCCTATTACTTGTCGTCATCGTCCTTGTAGTCCTCCATTAACCGCAACCAGTACTCCCAGAACCTGGGCA-3′, were phosphorylated with T4 polynucleotide kinase (Takara), mixed, and then annealed by heating to 98°C for 20 sec and cooling gradually to room temperature. The product was ligated into the NdeI/BamHI-digested GB1-fusion co-expression vector [31], in which the His tag was substituted with a HAT tag in advance. The resulting plasmid was used as a template for PCR using the primers: 5′- ATATGCCGCACCATGGGCAAAGATCATCTGATCCACAATG-3′ and 5′-CCCAGGTTCTGGGAGTACTGGTTGCGGTTAATGGAGGGTGGTGGTGAAAACCTGTACTTCCAGGGTATGTCTGTACCTACTGATGGTGC-3′.

On the other hand, the MDM2^12–108^ gene was amplified from the pCMV-MDM294-CBPzz plasmid [23] by PCR using the primers: 5′-ATGTCTGTACCTACTGATGGTGC-3′ and 5′-GTATGCCTCGAGCTATTAACCCATTTGCTGTCCACCAGTCATGCTAGCCATCATGGTATATATTTTCCTGTGCTCTTTC-3′.

**Figure 1:**
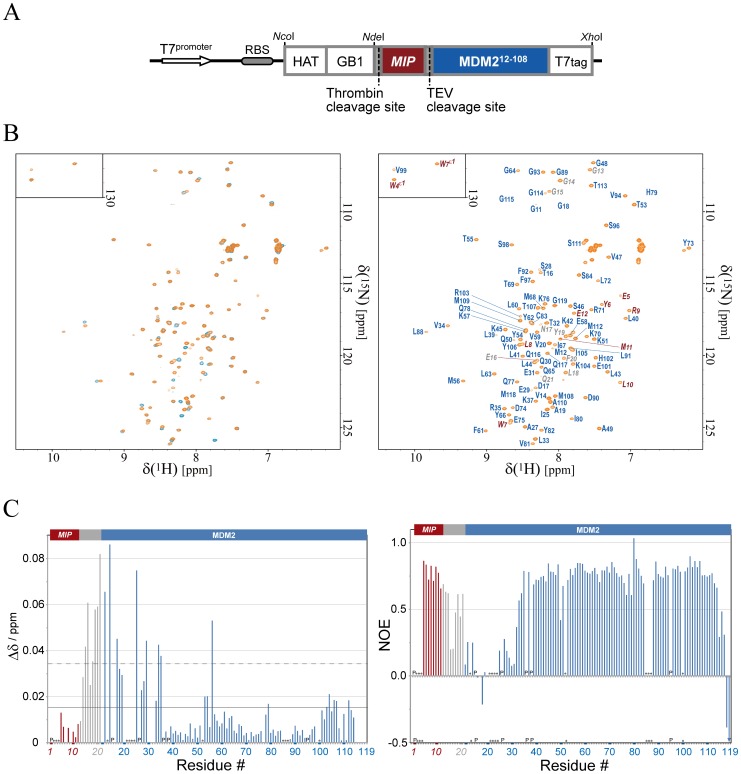
NMR analysis of the MIP-MDM2 fusion and MIP:MDM2 complex. (**A**) Schematic diagram of the HAT-GB1 fused MIP-MDM2-T7tag protein expression plasmid. The T7 promoter, ribosome binding site (RBS), restriction enzyme sites, and protease cleavage sites are also indicated. (**B, left**) Shown in orange is the 2D ^1^H-^15^N HSQC spectrum of the MIP-MDM2-T7tag linked protein (MIP-MDM2) and in cyan the MIP:MDM2-T7tag complex (MIP:MDM2 complex). (**B, right**) 2D ^1^H-^15^N HSQC spectrum of MIP-MDM2. Signals are labeled with the residue number and a one-letter amino acid code: The MIP, TEV cleavage site, and MDM2-T7tag portions are colored brown, gray, and blue, respectively. (**C, left**) Chemical shift differences of the corresponding signals in Fig. 1 (B, left). The chemical shift difference, Δδ, was determined as Δδ =  [(Δδ_H_)^2^+ (Δδ_N_/6.5)^2^]^1/2^, where Δδ_H_ and Δδ_N_ are the chemical shift differences for HN and ^15^N, respectively. The mean value and the mean value +1SD are shown by solid and dashed lines, respectively. (**C, right**) Steady-state ^1^H-^15^N NOE values are shown for MIP-MDM2. “P”s indicate proline residues and asterisks indicate residues whose ^1^H-^15^N resonance was not assigned.

The two PCR products were mixed and used as a template for overlap extension PCR with the primers: 5′-ATATGCCGCACCATGGGCAAAGATCATCTGATCCACAATG-3′ and 5′-GTATGCCTCGAGCTATTAACCCATTTGCTGTCCACCAGTCATGCTAGCCATCATGGTATATATTTTCCTGTGCTCTTTC-3′. Finally, the PCR product was subcloned into the NcoI/XhoI-digested pET15b plasmid to obtain the pMIP-MDM2 plasmid, which produces the HAT-GB1-MIP-MDM2-T7tag fusion protein (Figure 1A).

### Protein expression and purification

*Escherichia coli* strain BL21 (DE3) codon-plus was transformed with pMIP-MDM2. Cells were grown in LB containing 100 µg/mL ampicillin at 37°C to an optical density (OD_600_) of 0.6. After centrifugation at 2,500 *g* for 5 min at 4°C, the pellets were washed with a 140 mM NaCl aqueous solution. The washed pellets were resuspended in M9 minimal medium containing either 0.2 g/L ^15^N-NH_4_Cl (ISOTEC) or 1 g/L ^15^N-NH_4_Cl and 5 g/L ^13^C-glucose (ISOTEC) as nitrogen and carbon sources. The former and latter conditions were used to obtain ^15^N-single labeled and ^15^N/^13^C-double labeled proteins, respectively. The cells were grown at 30°C overnight to an OD_600_ of 0.7, and then protein expression was induced with 0.4 mM IPTG, followed by further incubation at 37°C for 4 h. The harvested cells were resuspended in lysis buffer (20 mM Tris-HCl, pH 7.6, 300 mM NaCl). After the addition of a protease inhibitor cocktail (Sigma), sonication (15 min×4 cycles) and centrifugation (2,500 *g* at 4°C for 20 min) were performed. The collected supernatant was loaded onto a TALON Metal Affinity Resin column (Clontech). After washing with 100 column volumes of lysis buffer, protein was eluted with lysis buffer containing 250 mM imidazole. The obtained protein was dialyzed against the thrombin cleavage buffer (20 mM Tris-HCl, pH 8.0, 150 mM NaCl, 250 mM CaCl_2_) and then treated with 2 units/mg protein of thrombin at room temperature for 16 h. The protein solution was passed through the TALON Metal Affinity Resin column for the second time. The HAT-GB1 tag was retained on the column. Finally, the MIP-MDM2 fusion protein, which was collected in the flow through fraction, was further purified by size-exclusion chromatography on a Superdex 75 10/300 GL column (GE Healthcare) in lysis buffer. The fractions containing the fusion protein were pooled and concentrated to about 10 mg/mL using an Amicon Ultra-4 (Millipore). Finally, three NMR samples: each containing 150 µM ^15^N-labeled MIP-MDM2 fusion, ^15^N-labeled 150 µM MIP:MDM2 complex, and 650 µM ^13^C,^15^N-labeled MIP-MDM2 fusion; all dissolved in 20 mM Tris-HCl, pH 7.6, 300 mM NaCl, and 5% ^2^H_2_O, were prepared.

### NMR spectroscopy

All NMR data were collected at 298 K on a Bruker AVANCE 600 MHz NMR spectrometer equipped with a cryogenic probe. NMR spectra were processed with NMRPipe/NMRDraw [32]. Spectral analysis was performed with Kujira 0.984 [33], a program suite for interactive NMR analysis working with NMRView [34], according to the method described previously [35]. The backbone and side chain ^1^H, ^15^N, and ^13^C resonances of MIP-MDM2-T7tag were assigned by means of standard double- and triple-resonance NMR experiments [36], [37], and were deposited in BioMagResDB (BMRB ID: 11569). Distance restraints were derived from three-dimensional (3D) ^15^N-edited and ^13^C-edited nuclear Overhauser effect spectroscopy (NOESY)-HSQC spectra, each being measured with a mixing time of 80 msec. To determine the steady-state ^1^H-^15^N NOE value of MIP-MDM2-T7tag, an enhanced-sensitivity experiment was performed using the standard method with the parameters described previously [35], [38]. The spectra were analyzed with Sparky [39] as described previously [35].

### Structure calculations

Structure calculations for MIP-MDM2-T7tag were performed using CYANA 2.1 [40]–[42], with the standard CYANA simulated annealing schedule and 40,000 torsion angle dynamics steps per conformer, starting with 200 randomized conformers. The 40 conformers exhibiting the lowest final CYANA target function values were further refined with AMBER12 [43], using the AMBER 2003 force field and a generalized Born model, as described previously [35]. The force constants for distance, torsion angle, and ω angle restraints were set to 32 kcal mol-1 Å^−2^, 60 kcal mol^−1^ rad^−1^, and 50 kcal mol^−1^ rad^−2^, respectively. The 20 conformers that were most consistent with the experimental restraints were then used for further analyses. The final structures were validated and visualized by using the Ramachandran plot web server [44] and software CHIMERA [45], [46]. Detailed experimental data and structural statistics are summarized in Table 1. The final ensembles of 20 conformers were deposited in the Protein Data Bank (PDB ID: 2RUH).

**Table 1:** Structural Statistics for MIP-MDM2.

NMR restraints	
Distance restraints	
Total NOE	1823
Intra-residue	568
Inter-residue	
Sequential (|i–j| = 1)	387
Medium-range (1<|i–j| <5)	365
Long-range (|i–j| ≥5)	503
Hydrogen bonds restraints a	34
Dihedral angle restraints a	
φ and ψ	3/3
χ^1^ and χ^2^	21/16
Structure statistics (20 conformers)	
CYANA target function (Å^2^)	0.29
Residual NOE violations	
Number >0.1 Å	3
Maximum (Å)	0.37
Residual dihedral angle violations	
Number >5°	0
Maximum (°)	0.83
AMBER energies (kcal/mol)	
Mean AMBER energy	−3612
Mean restraints violation energy	5.44
Ramachandran plot statistics (%) b	
Residues in most favored regions	82.3
Residues in additionally allowed regions	15.7
Residues in generously allowed regions	1.8
Residues in disallowed regions	0.2
Average R.M.S.D. to mean structure (Å) c	
Protein backbone	0.53
Protein heavy atoms	1.28

a Used only in CYANA calculations.

b Calculated with the Ramachandran plot server at the Indian Institute of Science.

c For residues Phe3-Glu12 of MIP and Leu33-Gly114 of MDM2.

### Surface plasmon resonance (SPR) analysis

Binding kinetics were determined by SPR with a Biacore 3000. All experiments were performed at 25°C using HBS-EP buffer (10 mM HEPES–NaOH, pH 7.4, 150 mM NaCl, 3 mM EDTA, 0.005% Tween-20). Biotinylated LC-biotin-MIP (PRFWEYWLRLME, 2,065 Da), LC-Biotin-DI (LTFEHYWAQLTS, 1,835 Da), and LC-Biotin-p53 (17-28 amino acid residues, ETFSDLWKLLPE, 1,817 Da) were chemically synthesized and immobilized on a streptavidin sensor chips, respectively. The measurements were performed with resonance units of 50.4 (MIP), 57.1 (DI) and 89.5 (p53^17–28^), and at a flow rate of 20 µl/min. The MDM2^7–300^ (7–300 amino acid residues) gene was amplified from the pDrive-MDM2 plasmid [23] by PCR using the primers: 5′-CACCATGTGCAATACCAACATGTCTG-3′ and 5′-CTTGAGCTCGAGATCTTCTTCAAATGAATCTGTATC-3′. The PCR product was subcloned into the pENTR/D-TOPO vector (Invitrogen). The resulting plasmid was recombined with the pDEST15 vector to generate a GST-MDM2^7–300^ expression construct (pDEST15-MDM2). pDEST15-MDMD2 was used for transformation of *E. coli* strain BL21 (DE3) codon-plus. The cells were grown in LB with 100 µg/ml ampicillin at 37°C until OD_600_ reached 0.7, induced with 1 mM IPTG for 5 h at 30°C, and then harvested by centrifugation. The pellets were resuspended in PBS supplemented with a protease inhibitor cocktail (Sigma), sonicated, and then centrifuged. The resulting supernatants were added to glutathione-Sepharose 4B (GE Healthcare), and then mixed on a rotator for 2 h at 4°C. The beads were washed with PBS and eluted with 50 mM Tris-HCl, pH 8.0, containing 50 mM gluthathione, followed by dialysis against PBS using Slide-A-Lyzer dialysis cassettes (Thermo Scientific) to obtain purified GST-MDM2^7–300^. To determine dissociation constants, two different concentrations (100 nM and 200 nM) of the purified GST-MDM2^7–300^ were injected. The injection periods for association and dissociation were 30 and 180 s, respectively. After each measurement, the chip surface was regenerated with 10 µl of Glycine 2.0 (Biacore). The binding data were analyzed with the 1∶1 Langmuir binding model in the BIAevaluation software ver. 4.1 (Biacore).

### CD spectroscopy

The CD spectra of chemically synthesized peptides (0.1 mM), i.e. MIP (PRFWEYWLRLME), DI (LTFEHYWAQLTS), and the p53 peptide (ETFSDLWKLLPE), were measured with a J-820 spectropolarimeter (Jasco) at 25°C in the presence of different concentrations of 2,2,2-trifluoroethanol (TFE, Wako). The light-path length used was 2 mm. The results were expressed as mean residue molar ellipticity [θ].

## Results and Discussion

### MIP binds to MDM2 with higher affinity than the known MDM2-binding peptides

We recently identified a highly optimized MDM2-binding peptide named MIP by performing selection of peptides that bind to MDM2, which was immobilized on IgG beads *via* the zz domain of protein A, from large random peptide libraries in two stages using mRNA display [23]. The sequence of MIP (^1^PRFWEYWLRLME^12^) is distinct from those of the corresponding peptides, p53^17–28^ peptide (^17^ETFSKLWKLLPE^28^), DI (^17^LTFEHYWAQLTS^28^) [14], and PMI (^1^TSFAEYWNLLSP^12^) [47], all of which share the Phe-Trp-Leu triad, which binds to the hydrophobic pockets of MDM2. According to the previous report on IC_50_ value determination by ELISA, DI inhibits the p53:MDM2 interaction 45-fold more effectively than the p53^17–28^ peptide, while PMI inhibits the p53:MDM2 interaction 2.2-fold more effectively than DI [47]. On the other hand, we showed that MIP exhibits a 29-fold higher IC_50_ value than that of DI for inhibition of the MDM:p53 interaction [23]. To further evaluate the affinities of MIP:MDM2, DI:MDM2, and p53^17–28^:MDM2 complexes, we conducted SPR analysis, obtaining dissociation constants (*K*_D_) of 18.4 nM, 210 nM, and 14.5 µM, respectively (Table 2). Thus, the rank order of the potency of these peptides for inhibiting the MDM2:p53 interaction appears to be MIP>PMI>DI>p53^17–28^. This finding prompted us to investigate the structural origin of the strong binding of MIP to MDM2.

**Table 2:** Kinetic analysis of MDM2 binding to MIP, DI or p53 peptide, as determined by SPR.

	k_a_ (1/Ms)^a^	k_d_ (1/s)^a^	K_A_ (1/M)	K_D_ (M)
MIP	1.20×10^5^	2.21×10^−3^	5.42×10^7^	1.84×10^−8^
DI	1.95×10^4^	4.09×10^−3^	4.77×10^6^	2.10×10^−7^
p53 peptide	411	5.98×10^−3^	6.88×10^4^	1.45×10^−5^

a the standard error for the kinetic parameters in each global fit was ≤1%.

### Structure determination of the MIP-MDM2 fusion protein by NMR

A HAT-GB1-MIP-MDM2-T7tag fusion protein was synthesized by using a bacterial overexpression system (Figure 1A). Our intention to use this fusion protein was to cost effectively and efficiently obtain ^15^N-single labeled and ^15^N/^13^C-double labeled MIP, so as to apply the standard double- and triple-resonance NMR experiments to the whole system. The HAT-GB1 portion was cleaved and eliminated during the purification steps, while the T7tag was left attached. The obtained MIP-MDM2-T7tag fusion protein (hereinafter referred to as the MIP-MDM2 fusion) was further treated with a protease and thereby cleaved at the C-terminal end of MIP, which resulted in generation of the MIP:MDM2-T7tag complex (referred to as the MIP:MDM2 complex). Comparison of the ^1^H-^15^N HSQC spectra of the MIP-MDM2 fusion and MIP:MDM2 complex showed the considerable similarity in their signal patterns (Figure 1B, left). Signal assignments of the ^1^H-^15^N HSQC spectra were performed (Figure 1B, right), and chemical shift differences between these two ^1^H-^15^N HSQC spectra were further analyzed, it being found that the Δδ values of all the residues are very small (Figure 1C, left). Despite the small Δδ values (all the values are less than 0.1 ppm), the residues in the flexible regions such as the linker portion attached to the C-terminus of MIP and the N-terminal region of MDM2 (see steady-state ^1^H-^15^N NOE values in Figure 1C, right), and some residues in the less flexible regions of MDM2 (T53, Y54, M56, H79, K104, Y106, and T107, which will be discussed later in this section) showed larger Δδ values. Thus, we concluded that the structures of the MIP-MDM2 fusion and MIP:MDM2 complex are similar if not the same, and decided to carry out the structural study using the MIP-MDM2 fusion.

**Figure 2:**
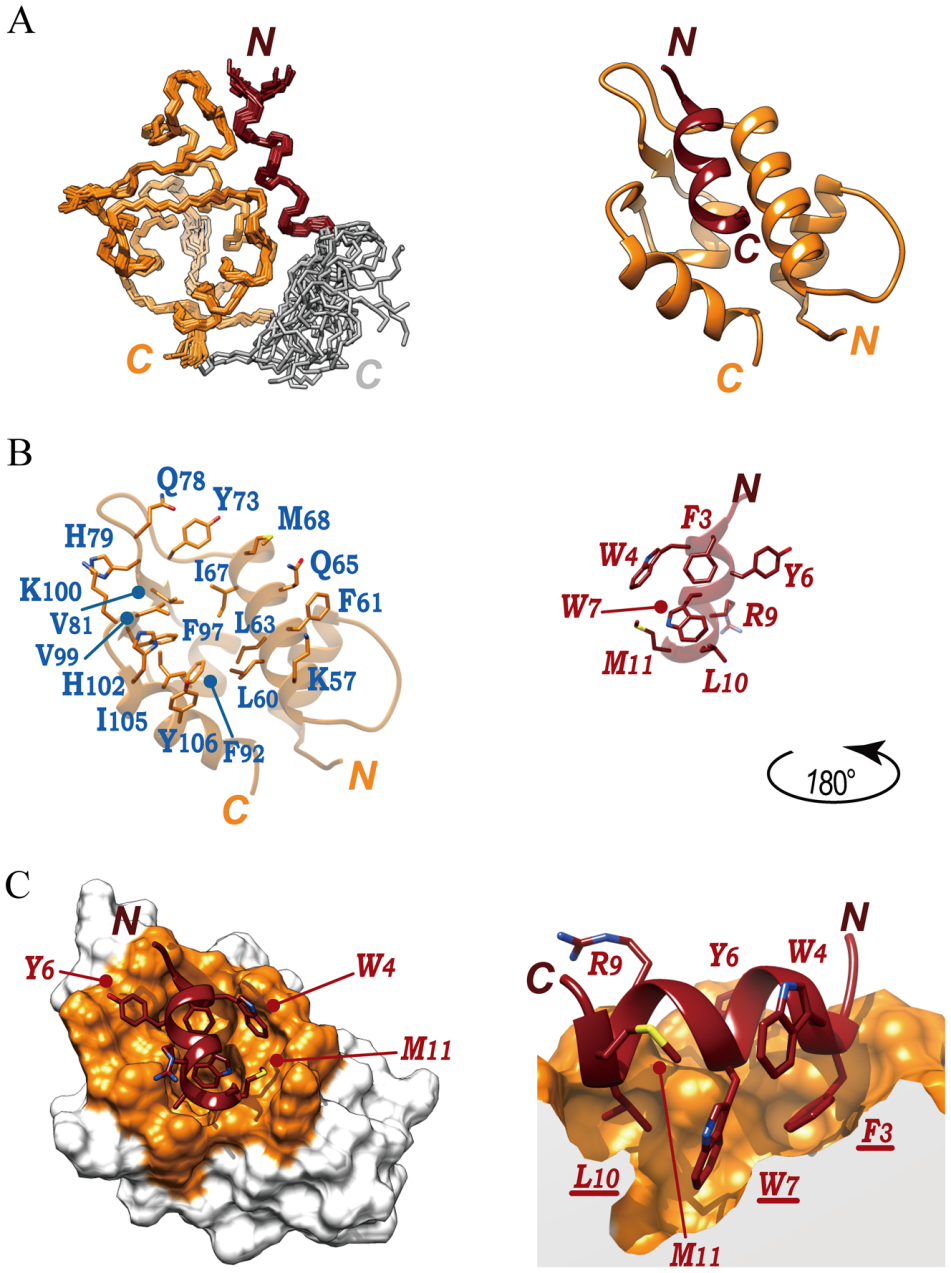
NMR solution structure of the MIP-MDM2 fusion. (**A, left**) Superpositioning of backbone heavy atoms of the 20 MIP-MDM2 three-dimensional structures. Residues R2-E12 for MIP, G13-Q22 for linker portion, and L33-G114 for MDM2-T7tag, which exhibited steady-state ^1^H-^15^N NOE values of >c.a. 0.5, are shown. (**A, right**) Ribbon representation of the lowest energy structure of MIP-MDM2, which is a 90° rotated view of Fig 2A, left. (**B, left**) MDM2 residues whose side chain atoms are within 3 Å from the side chain atoms of the MIP residues, are displayed. (**B, right**) MIP residues whose side chain atoms are within 3 Å from the side chain atoms of the MDM2 residues are displayed. MDM2 is viewed as in Fig. 2A, right, while MIP is rotated by 180° around the y-axis. The ribbons were de-emphasized by making them 50% transparent. (**C, left**) MIP sits on the concave surface of MDM2. MDM2 residues that are displayed in Fig. 2B are colored orange. Same view as in Fig. 2A, right. (**C, right**) A side view of MIP, which is focused on the Phe-Trp-Leu triad that is conserved among the MDM2-binding peptides. The MDM2 portion is shown as a sliced surface representation.

To determine the structure of the MIP-MDM2 fusion, NMR experiments, spectral analysis, and structural calculation were performed following the methods described previously [35]. The experimental restraints and structural statistics for the 20 lowest energy structures are summarized in Table 1, it being indicated that residues F3-E12 of MIP and L33-G114 of MDM2 adopt a well-defined structure, with an RMSD of 0.53 Å for the backbone atoms (Figure 2A). Although, some residues in the linker portion (G13, G14, G15, Y19, and Q21) showed higher steady-state ^1^H-^15^N NOE values (>0.5) in Figure 1C (right), we were not able to identify their inter-residue NOE signals in NOESY spectra. Thus, it is assumed that the mobility of some part of the linker portion might have been restricted by such as steric hindrance (Figure 2A, left). The MIP portion is composed of a single α-helix, F3-E12, while the MDM2-T7 portion comprises four α-helices: αA (P38-V47), αB (M56-K70), αC (L87-F92), and αD (H102-M112); and two short β-strands: β1 (I80-Y82) and β2 (S96-S98). It can be seen that the last one-third of αD, which corresponds to the N-terminal region of the T7tag, appears as less flexible (Figure 1C, right), but distant from the MIP binding site.

Among the aforementioned residues of MDM2 that showed larger Δδ values in Figure 1C (left), the residues T53, Y54, M56, H79, K104, Y106, and T107 are located in the structured regions, which is consistent with the steady-state ^1^H-^15^N NOE values (Figure 1C, right). As expected, the locations of the residues T53, Y54, M56, K104, Y106, and T107 are close to the linker portion. On the other hand, the location of H79 seemed far from the linker portion at a first glance. However, it turned out that the location of H79 is close to those of the residues K104, Y106, and T107 on the same surface of MDM2. Although, it is not possible to determine the position of the linker portion because it is flexible, all the residues that showed larger Δδ values in Figure 1C (left), including H79, are indeed seem to locate close to the linker portion.

An opened-up view of the MIP and MDM2 interface shows that the residues in close contact (intermolecular distances of up to 3 Å) are mostly hydrophobic (Figure 2B). MIP fits into the large concavity on the MDM2 surface (Figure 2C, left). The Phe-Trp-Leu triad of MIP, which is completely conserved among the MDM2-binding peptides, orients the side chains deep inside the hydrophobic pockets located at the center of the large concavity (Figure 2C, right). Additionally, three rather large hydrophobic residues, W4, Y6, and M11, of MIP fit on the hydrophobic patches along the rim of the large concavity of MDM2 (Figure 2C, left). These three residues are unique to MIP and the most probable candidates increasing the MIP′s affinity towards MDM2.

### Binding mode of MIP to MDM2

**Figure 3:**
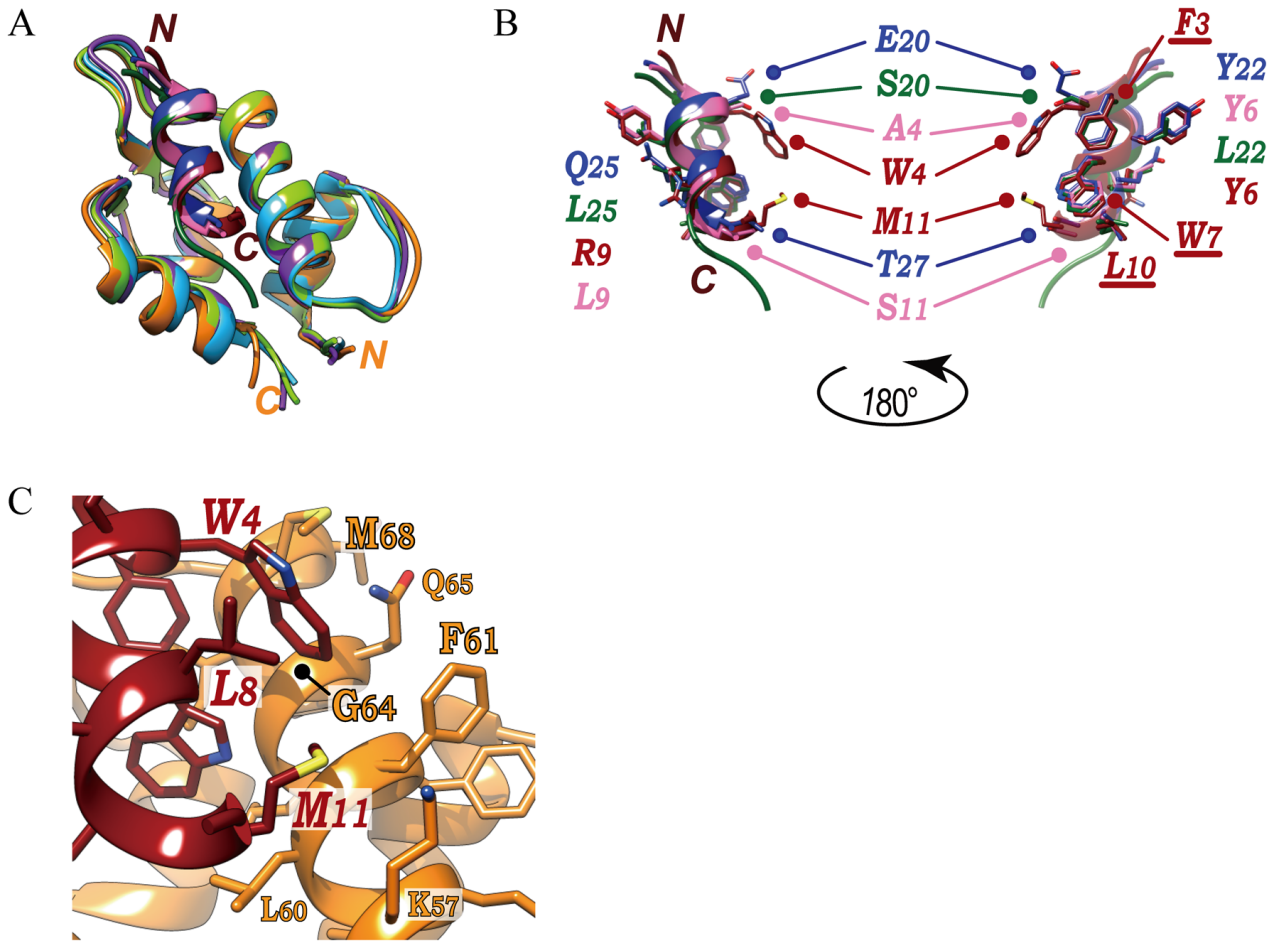
Comparison of the MDM2-binding modes between MIP and other peptides. (**A**) superpositions of MIP-MDM2 fusion (PDB ID = 2RUH), DI:MDM2 (3G03), PMI:MDM2 (3EQS), and p53 peptide:MDM2 (1YCR) in ribbons, viewed as in Fig. 2A, right. Each component is color coded: MIP (brown)-MDM2 (orange), DI (blue):MDM2 (cyan), PMI (pink):MDM2 (purple), and the p53 peptide (green):MDM2 (light green). (**B**) MIP, DI, PMI, and p53 peptide are presented as two opposite views. W4, Y6, and M11 of MIP cover the hydrophobic patches on the surface of MDM2, as shown in Fig. 2C, left. These residues and the Phe-Trp-Leu triad, and the corresponding residues in DI, PMI, and the p53 peptide are displayed as sticks. (**C**) A-zoomed-in view of the hydrophobic contacts formed around W4 and M11 of MIP. Fig. 3A, Fig. 3B (left), and Fig. 3C are views from the same direction as in Fig. 2A (right), Fig. 2B (left), and Fig. 2C (left).

The solution structure of the MIP-MDM2 fusion is very similar to the crystal structures of the p53^15–29^:MDM2 complex (RMSD of 0.74 Å between superimposed 80 C_α_ atoms), DI:MDM2 complex (RMSD of 0.95 Å between superimposed 81 C_α_ atoms), and PMI:MDM2 complex (RMSD of 0.79 Å between superimposed 79 C_α_ atoms) (Figure 3A). As expected, the side chains of the conserved Phe-Trp-Leu triad of MIP superimpose very well with those of DI, PMI, and the p53 peptide (Figure 3B). On the other hand, the lengths of the α-helices are in the order of MIP ≥DI≈PMI>p53 peptide, where the α-helix of MIP is either the same length as or one residue longer than those of DI and PMI, and two residues longer than that of the p53 peptide. Extension of the α-helix in MIP is achieved by M11. In the case of the p53 peptide, this position is occupied by proline, which is known as a breaker of an α-helical structure. Substitution of this proline to serine was shown to endow the p53 peptide with its α-helical nature [48]. The corresponding positions in DI and PMI are occupied by threonine (T27) and serine (S11), respectively. T27 of DI was seen to continue the helical turn and H-bonding pattern loosely [49]. It was briefly mentioned in the previous section that M11 of MIP covers a hydrophobic patch on the surface of MDM2. In detail, its side chain forms hydrophobic contacts with K57 and F61 of MDM2, which is supported by the observation of NOEs between the methyl H_ε_ of M11 and the side chains of K57 and F61. Thus, it seems that these hydrophobic contacts together with the backbone and side chain conformations of M11 are mutually stabilized.

The positions of the other aforementioned large hydrophobic residues, W4 and Y6, of MIP, which form hydrophobic contacts with MDM2, were also compared (Figure 3B). Interestingly, the position of Y6 in MIP is also occupied by tyrosine in DI (Y22) and PMI (Y6), but by leucine in P53 peptide (L22). Since tyrosine has a much larger side chain than leucine, MIP, DI, and PMI are able to fill the hydrophobic concavity on the MDM2 surface, while the corresponding space remains solvent-accessible in the p53 peptide:MDM2 complex. This additional interaction by tyrosine at this position and the fact that its side chain forms extensive hydrophobic contacts with H73 and K94 of MDM2 were indicated in the respective structures of DI:MDM2 and PMI:MDM2 complexes [47], [49]. Thus, tyrosine at this position is highly preferred by MDM2-binding peptides to achieve strong binding.

The role of the position of W4 in MIP exhibits distinct differences with those of the equivalent positions in DI, PMI, and p53 (Figure 3B). The side chain ring of W4 is sandwiched by the methyl groups of L8 of MIP and M68 of MDM2 in the so called CH-π interaction manner (Figure 3C). Additionally, the backbone of G64 and the plane surface of the side chain ring of F61 in MDM2, and the methyl group of MIP M11 surround the side chain ring of W4 (Figure 3C). The presence of these interactions is fully supported by NOEs. The residues in DI, PMI, and p53 at the equivalent position to W4 in MIP are E20, A4, and S20, respectively (Figure 3B). These residues seem not to undergo direct intermolecular interactions in their respective complex structures. Hence, W4 in MIP adds an extra intermolecular interaction on the surface of MDM2 and thereby contributes to stronger binding to MDM2.

### MIP preferentially forms α-helical structure in a hydrophobic environment

**Figure 4:**
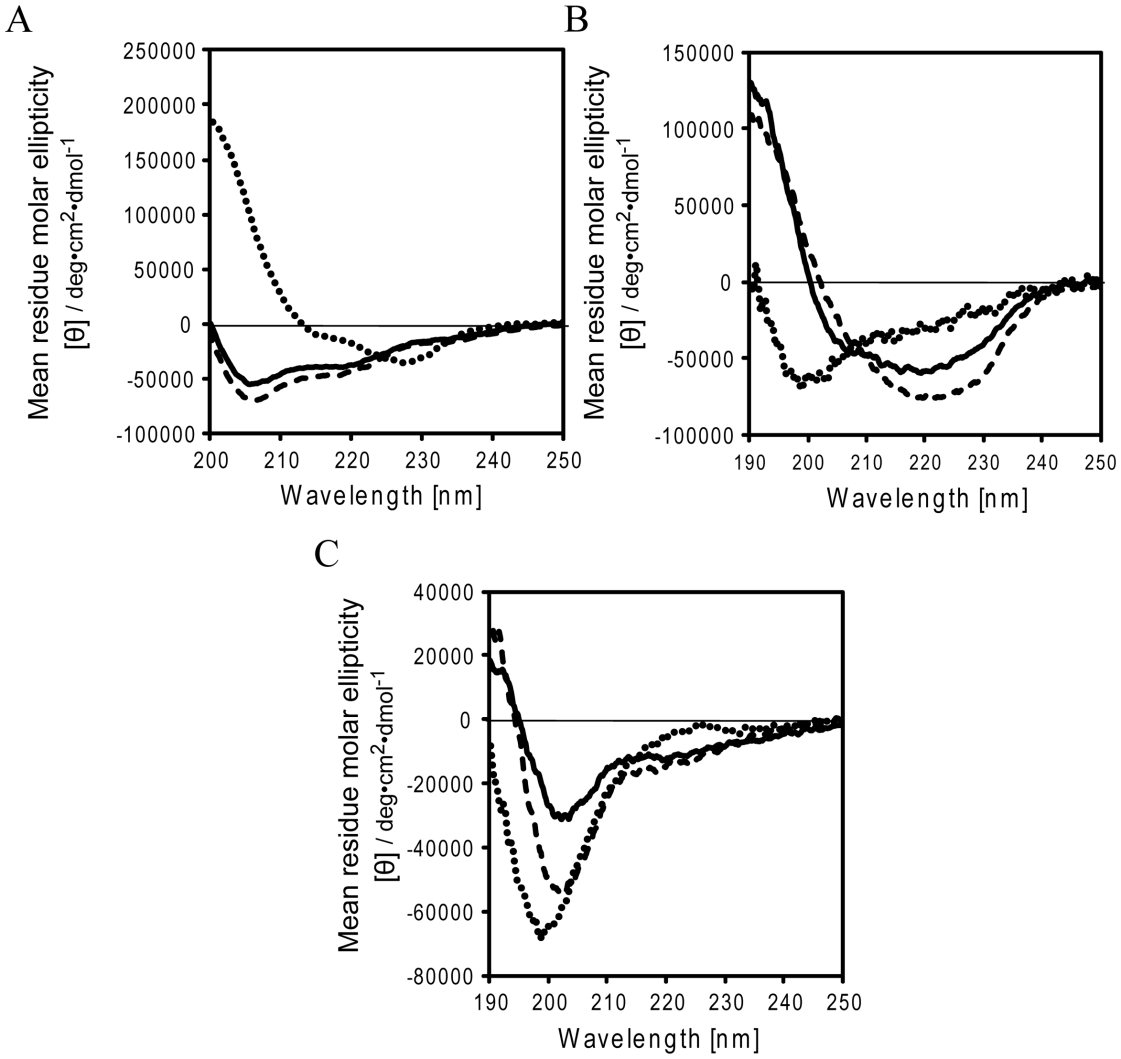
MIP preferentially forms α-helical structure in a hydrophobic environment. CD spectra of MIP (**A**), DI (**B**), and the p53 peptide (**C**) with different concentrations: 0% (solid line), 30% (dashed line), and 50% (dotted line) of TFE.

A large number of intrinsically disordered regions (or intrinsically unstructured domains), which become structured only during binding to the target (i.e., coupled folding and binding), have already been identified in nature [50]. The N-terminal region of p53 is intrinsically unstructured in solution [50] but, however, it folds into an amphipathic helical structure upon binding to its target protein, MDM2 [20]. As described above, the NMR data analysis suggested that hydrophobic contacts between MIP and MDM2 play critical role to their strong binding. A hydrophobic environment generally stabilizes the formation of secondary structures of peptides and proteins [51]–[53]. Therefore, we examined the influence of a hydrophobic environment on the structure formation of MIP. Circular dichroism (CD) spectra of MIP, DI, and the p53 peptide were measured in the presence of different concentrations of a 2,2,2-trifluoroethanol (TFE), which mimics a partial hydrophobic environment (Figure 4).

The CD spectrum of the p53 peptide in the absence of TFE showed a characteristic spectral pattern for an unstructured peptide (Figure 4C). The presence of TFE hardly affected the spectral pattern of the p53 peptide, which indicates that a hydrophobic environment is not sufficient to promote the structure formation of the p53 peptide. On the other hand, TFE caused distinct changes in the CD spectral patterns of MIP and DI, respectively (Figure 4A, B). In particular, the CD spectrum of MIP in the presence of TFE exhibited a characteristic pattern of α-helical structure. A characteristic minimum at 227 nm in the CD spectrum of MIP in the absence of TFE may be due to W4 and/or W7 of MIP [54], [55]. The CD spectrum of DI also showed a characteristic pattern of an unstructured peptide in the absence of TFE, however, the presence of TFE converted it to a pattern of a mixture of secondary structure (Figure 4B). Thus, strong binding of MIP with MDM2 is not only due to the enlarged binding interface, but supposedly also due to the preferred formation and stabilization of the α-helical structure in MIP in the hydrophobic environment.

## Conclusions

In this study, we investigated the interaction between MDM2 and MIP, the optimal 12-mer peptide that we had screened and identified from random peptide libraries using the *in vitro* virus (mRNA display) method, through NMR structure determination. MIP utilized not only the sequentially and functionally conserved Phe-Trp-Leu triad to fill the hydrophobic pockets of MDM2 but also the solvent-exposed W4, Y6, and M11 to enlarge the binding interface and to cover the hydrophobic surface patches that surround the hydrophobic pockets of MDM2. The first case of involvement of W4 and M11 in binding with MDM2 was confirmed structurally in this study. Significance of Y6 and M11 in binding with MDM2 was also supported by previous mutation experiments [23]. The structural information we obtained here provides important clues for designing small molecule inhibitors of the MDM2:p53 binding. Therefore, we are going to approach design of novel small molecule inhibitors of the MDM2:p53 binding through docking simulation based on this structural information. Currently known small molecule inhibitors, Nutlin-3 for example [16], have functional groups that only fit into the hydrophobic pockets, however, our results suggest that the hydrophobic surface patches surrounding the hydrophobic pockets should also be considered. Thus, the structure of MIP in the complex should be suitable as a template for designing a new small molecular inhibitor. Furthermore, for development of drugs utilizing MIP itself, we are constructing novel fusion proteins in which MIP was connected with transmembrane amino acid sequences and exploring an efficient delivery system of MIP to inside the cell.
